# Operation for Hare-Lip

**Published:** 1844-05-18

**Authors:** 


### OPERATION FOR HARE-LIP.

Mr, Fergusson's opinion is, that under all circumstances, the best period for operating is immediately after suckling, provided the patient is otherwise in good health, and does not suffer from teething. It is usually a very safe operation at all periods of life.—*Ibid.*

